# Melanocortin Receptor 4 (MC4R) Signaling System in Nile Tilapia

**DOI:** 10.3390/ijms21197036

**Published:** 2020-09-24

**Authors:** Tianqiang Liu, Yue Deng, Zheng Zhang, Baolong Cao, Jing Li, Caiyun Sun, Zhixing Hu, Jiannan Zhang, Juan Li, Yajun Wang

**Affiliations:** Key Laboratory of Bio-Resources and Eco-Environment of Ministry of Education, College of Life Sciences, Sichuan University, Chengdu 610065, China; bamboo.foxer@gmail.com (T.L.); 2017222040095@stu.scu.edu.cn (Y.D.); zhang95424@outlook.com (Z.Z.); cbl-scu@outlook.com (B.C.); 2016222040084@stu.scu.edu.cn (J.L.); suncaiyun1237@gmail.com (C.S.); zhixinghu855@gmail.com (Z.H.); lijuanhk@gmail.com (J.L.)

**Keywords:** Nile tilapia, MC4R, MRAP2, ACTH, AgRP, α-MSH, β-MSH

## Abstract

The melanocortin receptor 4 (MC4R) signaling system consists of MC4R, MC4R ligands [melanocyte-stimulating hormone (MSH), adrenocorticotropin (ACTH), agouti-related protein (AgRP)], and melanocortin-2 receptor accessory protein 2 (MRAP2), and it has been proposed to play important roles in feeding and growth in vertebrates. However, the expression and functionality of this system have not been fully characterized in teleosts. Here, we cloned tilapia *MC4R*, *MRAP2b*, *AgRPs* (*AgRP*, *AgRP2*), and *POMCs* (*POMCa1*, *POMCb*) genes and characterized the interaction of tilapia MC4R with MRAP2b, AgRP, α-MSH, and ACTH in vitro. The results indicate the following. (1) Tilapia MC4R, MRAP2b, AgRPs, and POMCs share high amino acid identity with their mammalian counterparts. (2) Tilapia MRAP2b could interact with MC4R expressed in CHO cells, as demonstrated by Co-IP assay, and thus decrease MC4R constitutive activity and enhance its sensitivity to ACTH_1-40_. (3) As in mammals, AgRP can function as an inverse agonist and antagonist of MC4R, either in the presence or absence of MRAP2b. These data, together with the co-expression of *MC4R*, *MRAP2b*, *AgRPs,* and *POMCs* in tilapia hypothalamus, suggest that as in mammals, ACTH/α-MSH, AgRP, and MRAP2 can interact with MC4R to control energy balance and thus play conserved roles in the feeding and growth of teleosts.

## 1. Introduction

There has been increasing evidence showing that the melanocortin receptor 4 (MC4R) signaling system is crucial in the control of food intake and energy balance in the hypothalamus of vertebrates [1,2,3,4,5,6]. In mammals and birds, this signaling system consists of MC4R (a G protein-coupled receptor), MC4R ligands [α-melanocyte-stimulating hormone (α-MSH); adrenocorticotropin (ACTH), and agouti-related peptide (AgRP)], and melanocortin receptor 2 accessary protein 2 (MRAP2) [2,5]. α-MSH and ACTH are derived from the proopiomelanocortin (POMC) precursor and can activate MC4R and increase intracellular cAMP levels, while AgRP can act either as an antagonist to block α-MSH/ACTH action on MC4R, or as an inverse agonist to decrease the basal constitutive activity of MC4R in the absence of α-MSH/ACTH [1,7,8]. MRAP2 is a single-pass transmembrane protein and can form an antiparallel homodimer [9]. It can not only decrease the basal constitutive activity of MC4R, but also modulate its sensitivity and selectivity toward its ligands [2,5].

It is clear that in mammals, all the components of the MC4R signaling system are expressed in the hypothalamus and play a key role in the regulation of food intake, growth, and energy balance. α-MSH/ACTH secreted by POMC neurons can suppress the appetite via MC4R, while AgRP secreted by AgRP neurons can increase food intake by decreasing the constitutive activity of MC4R or antagonizing the action of α-MSH on MC4R [1]. Loss-of-function mutations in MC4R lead to obesity in mice [10]. Transgenic mice over-expressing AgRP show increased obesity, food intake, and hyperinsulinemia [11,12]. The mutation of MRAP2 in humans and mice also causes early-onset severe obesity [2]. All these findings support the notion that MC4R, α-MSH/ACTH, AgRP, and MRAP2 can form a complex interaction network to regulate the feeding, energy balance, and growth of animals [2,5].

As in mammals and birds, *MC4R*, *AgRP*, *POMC,* and *MRAP2* have also been identified in some teleost species [7,13,14,15], and these molecules likely play similar roles in feeding, growth, and obesity within the hypothalamus [16,17,18]. The intracerebroventricular (icv) or intraperitoneal (ip) injection of MC4R agonists can decrease food intake in goldfish [19], zebrafish [3], and coho salmon [20], while transgenic zebrafish over-expressing *AgRP* display obesity, increased linear growth, and adipocyte hypotrophy [17]. Moreover, in zebrafish, MRAP2a/MRAP2b can also interact with MC4R, regulate food intake and growth, and modulate the sensitivity of MC4R to α-MSH/ACTH [3,4]. All these findings imply that the MC4R signaling system plays critical roles in food intake and growth, similar to that in mammals and birds [5,6,21].

Despite the presence of the MC4R signaling system in teleosts, this system varies significantly between teleosts and mammals/birds. For instance, a single copy of each component (*MC4R, POMC, AgRP, MRAP2*) is present in mammalian and avian MC4R systems [1,5]. In contrast, two to three copies of *POMC* genes (e.g., *POMCa1*, *POMCa2,* and *POMCb* in barfin flounder) [22,23], two copies of *AgRP* genes (e.g., *AgRP* and *AgRP2* in zebrafish) [24,25], and one to two copies of *MRAP2* (e.g., *MRAP2a* and *MRPA2b* in zebrafish) [3,4] have been identified in teleosts. This difference raises a question of whether all the genes are expressed in the hypothalamus and function in a way similar to those in mammals and birds. Therefore, using tilapia as an animal model, our present study aimed to (1) identify and clone the genes of the MC4R signaling system; (2) examine whether MC4R can interact with MRAP2 (named MRAP2b in this study) and respond to their ligands, including the POMC-derived peptides (α-MSH, β-MSH, and ACTH_1-40_) and AgRP; and (3) examine the expression of the MC4R signaling system in the hypothalamus. The results from this study will provide comparative insights into the conserved roles of the MC4R signaling system in feeding and growth across vertebrates.

## 2. Results

### 2.1. Cloning of Tilapia MC4R, MRAP2b, POMCa1, POMCb, AgRP, and AgRP2 Coding Regions

**Figure 1:**
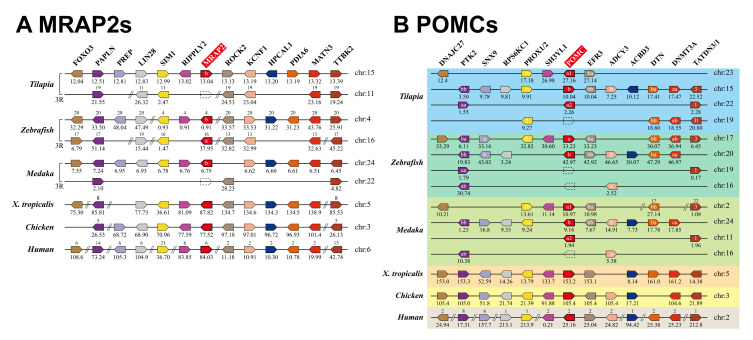
Synteny analysis of *MRAP2* (**A**), *POMC* (**B**), and their neighboring genes in Nile tilapia, zebrafish, Japanese medaka, *Xenopus tropicalis*, chickens, and humans. Orthologs are aligned in the same pentagon with the same color. The gene names are placed on top of the pentagons, and *MRAP2* (or *POMC*) gene was highlight with red background. Chromosome (Chr.) numbers are represented above the genes or listed on the right, and the locations (in megabase, Mb) on the chromosomes are shown below the genes based on the information from ENSEMBL databases. In some teleost species (e.g., zebrafish), two copies of *MRAP2* generated by the 3rd round of genome duplication (3R) were designated as *MRAP2a* and *MRAP2b*, respectively. In tilapia and medaka, three *POMCs* (*POMCa1*, *POMCa2*, and *POMCb*) were identified, which were likely originated by the whole genome duplication events during vertebrate evolution.

To determine whether the MC4R signaling system exists in tilapia, we searched the tilapia genome using ortholog genes in humans and zebrafish. Finally, we identified seven genes of the MC4R signaling system in tilapia, including one *MC4R*, one *MRAP2*, three *POMCs*, and two *AgRP* genes. According to the synteny analysis shown in Figure 1 and Figure S1 and nomenclature in other teleosts (e.g., barfin flounder, zebrafish), we designated them as tilapia *MC4R*, *MRAP2b*, *POMCa1*, *POMCa2*, *POMCb*, *AgRP,* and *AgRP2*, respectively.

**Figure 2:**
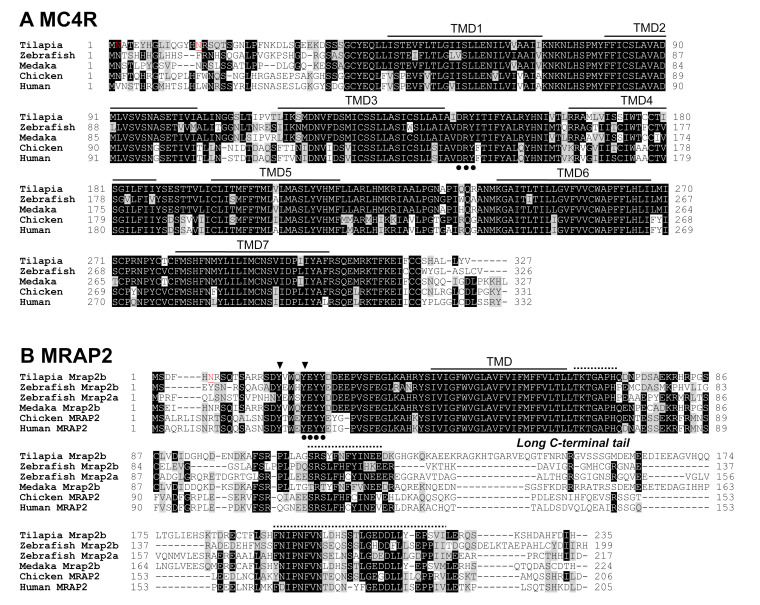
Amino acid sequence alignment of melanocortin receptor 4 (MC4R) and melanocortin-2 receptor accessory protein 2 (MRAP2). (**A**) Amino acid alignment of tilapia MC4R (MT500790) with that of zebrafish (NP_775385), medaka (XP_004081243), chickens (NP_001026685), and humans (NP_005903). Horizontal lines mark the seven putative transmembrane domains (TMD1–7). Two *N*-glycosylation sites are underlined and highlighted in red. The DRY motif is highlighted with dark dots. (**B**) Alignment of tilapia MRAP2b (MT500791) with that of zebrafish (mrap2a: XP_001342923, mrap2b: XP_005168578), medaka (XP_004083625), chickens (NP_001307836) and humans (NP_612418). Horizontal line marks the transmembrane domains (TMD). An *N*-glycosylation site is underlined and highlighted in red. Dots/arrowheads indicate the residues conserved in MRAP2s across vertebrates. The three conserved regions in the long C-termini of vertebrate MRAP2s are indicated by dotted lines.

Using RT-PCR, we further amplified and cloned the coding regions of *MC4R*, *MRAP2b*, *POMCa1*, *POMCb*, *AgRP,* and *AgRP2* from tilapia brain tissue. Sequence analysis revealed that tilapia MC4R (accession no.: MT500791) has seven typical transmembrane regions (TMD1–7) (Figure 2A). Similar to mammalian MC4R, tilapia MC4R has a conserved glycosylation site at its N-terminus and a conserved ‘DRY’ motif near the third transmembrane region. Sequence alignment showed that tilapia MC4R shares high amino acid sequence identities with zebrafish (78%), medaka (89%), amazon molly (93%), and zebra mbunda (99%), and it has a comparatively low identity with humans and mice (≈70%).

Tilapia *MRAP2b* cDNA (accession no: MT500790) encodes a protein of 235 amino acids. It shares a high degree of amino acid identities with that of medaka (79%), amazon molly (82%), and zebra mbunda (98%), but a comparatively low identity with that of zebrafish MRAP2b (50%) and MRAP2a (56%), chickens (49%), humans (54.0%), and mice (51%). Similar to human MRAP2, tilapia MRAP2b contains a glycosylation site at the N-terminus, which is a key motif (LKAHRYS) [26] for the formation of an antiparallel homodimer, and a long C-terminal tail containing three conserved regions across vertebrates (Figure 2B) [5].

**Figure 3:**
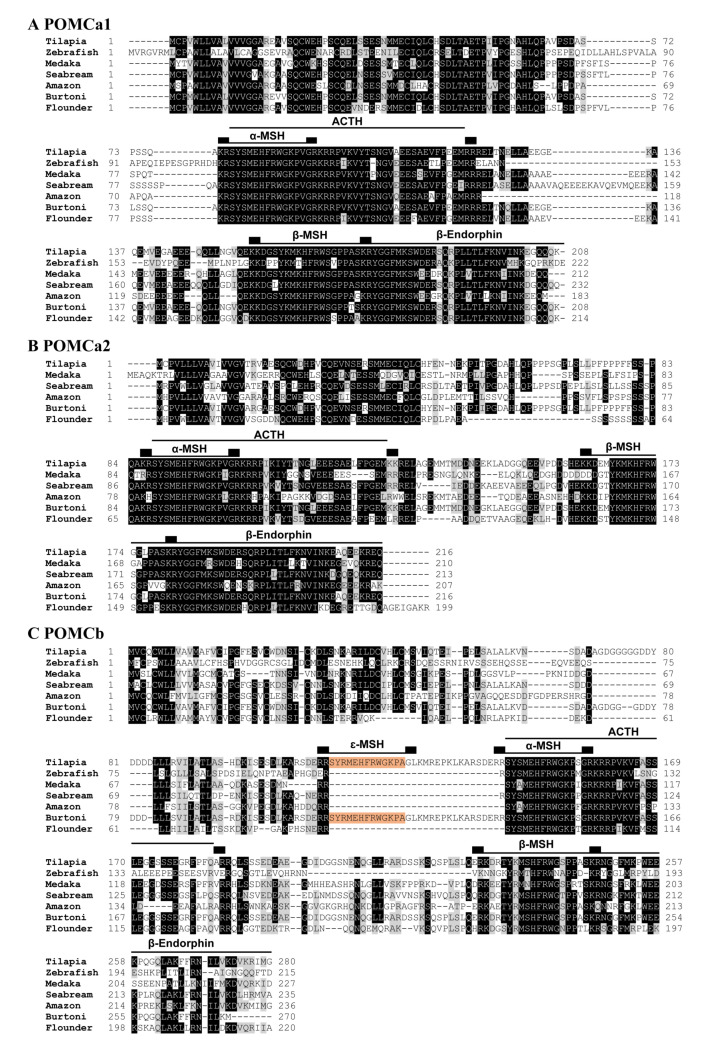
Alignment of POMC amino acid sequences in teleosts. (**A**) Amino acid alignment of the cloned tilapia POMCa1 (MT740811) with that of zebrafish (NP_852103), Japanese medaka (XP_004066504), gilthead seabream (AEI28997), amazon molly (XP_016536498), Burton’s mouthbrooder (NP_001273262), and barfin flounder (BAB18468). (**B**) Amino acid alignment of predicted tilapia POMCa2 (XP_003458632) with that of Japanese medaka (XP_023815525), gilthead seabream (AEI28996), amazon molly (XP_007569132), Burton’s mouthbrooder (NP_001302480), and barfin flounder (BAB18467). (**C**) Amino acid alignment of the cloned tilapia POMCb (MT740812) with that of zebrafish (NP_001076520), Japanese medaka (XP_004066504), gilthead seabream (AEI28997), amazon molly (XP_007552912), Burton’s mouthbrooder (NP_001273211), and barfin flounder (BAB18469). Horizontal lines indicate α-MSH/ACTH/β-MSH/β-endorphin peptides. The ɛ-MSH in tilapia and Burton’s mouthbrooder are masked in orange. Black boxes indicate endoproteolytic cleavage sites.

Two *POMC* genes were cloned from Nile tilapia: *POMCa1* (MT740811) and *POMCb* (MT740812). Each tilapia POMC precursor is predicted to generate α-MSH, ACTH, and β-MSH after cleavage at the dibasic residues (Figure 3). Moreover, we note the presence of an ε-MSH in the tilapia POMCb precursor. Although tilapia *POMCa2* cDNA was not cloned in this study, similar to the POMCa1 precursor, the predicted tilapia POMCa2 precursor (XP_003458632) seems to be capable of producing α-MSH, β-MSH, ACTH, and β-endorphin (Figure 3C). Unlike mammalian POMC, tilapia POMCs cannot produce ɤ-MSH [27]. The full list of POMC-derived peptides in tilapia is given in Table 1.

**Figure 4:**
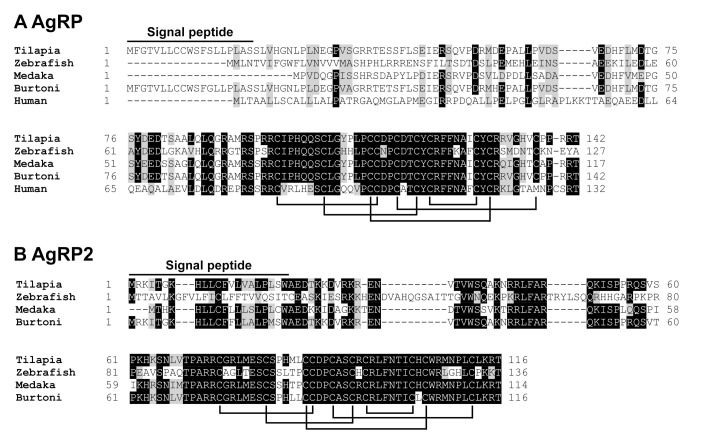
Alignment of AgRP and AgRP2 in teleosts. (**A**) Amino acid alignment of tilapia AgRP (MT740813) with that of zebrafish (NP_001314941), Japanese medaka (FAA00766.1), Burton’s mouthbrooder (XP_005929271), and humans (NP_001663). (**B**) Amino acid alignment of tilapia AgRP2 (MT740814) with that of zebrafish (NP_001258220), Japanese medaka (XP_004078940), and Burton’s mouthbrooder (XP_005939107). Signal peptides are marked by horizontal lines. Lines joining cysteine residues indicate the intra-molecular disulfide bonds.

As in zebrafish and medaka, two *AgRP* genes, namely *AgRP* (MT740813) and *AgRP2* (MT740814), were identified and cloned in tilapia. The amino acid sequence identity shared between mature tilapia AgRP (142 aa) and AgRP2 (116 aa) is low (23.4%). However, tilapia AgRP and AgRP2 share high amino acid sequence identities with zebrafish AgRP (51%) and AgRP2 (45%) respectively, with the five putative disulfide bonds fully conserved across vertebrates (Figure 4) [24,25].

### 2.2. Functionality of Tilapia MC4R in the Absence or Presence of MRAP2b

To detect whether α-MSH/ACTH can activate tilapia MC4R, we used synthetic tilapia α-MSH, β-MSH, and ACTH_1-40_ (derived from tilapia POMCa1 precursor) to treat CHO cells transiently expressing tilapia MC4R, and then, the receptor-stimulated cAMP/PKA signaling pathway was monitored by a pGL3-CRE-luciferase reporter system, as established in our previous study [5,28].

**Figure 5:**
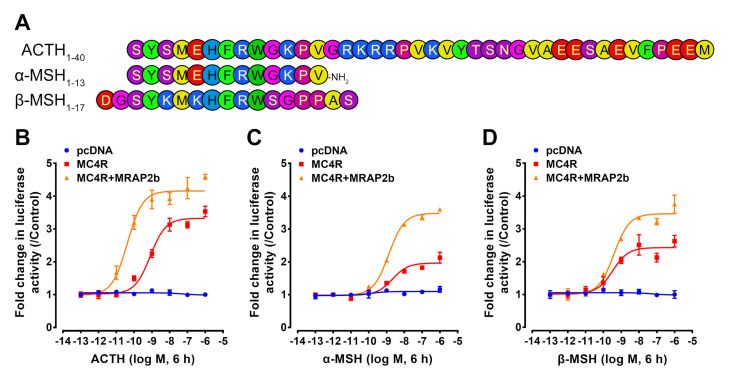
Effect of tilapia ACTH, α-MSH, and β-MSH in activating MC4R in the absence or presence of MRAP2b. (**A**) Sequences of tilapia ACTH, α-MSH, and β-MSH used in this study. The colors indicate amino acids of different biochemical properties. (**B**–**D**) Effect of ACTH (**B**), α-MSH (**C**), or β-MSH (**D**) in activating tilapia MC4R expressed in CHO cells, in the absence or presence of MRAP2b, as monitored by the pGL3-CRE-luciferase reporter system. The cells transfected by pcDNA3.1 (+) empty vector were used as the negative control. Each data point represents the mean ± SEM of 3 replicates (*n* = 3).

As shown in Figure 5, tilapia α-MSH, β-MSH, and ACTH could activate MC4R expressed in CHO cells and stimulate the luciferase activity dose-dependently. The EC_50_ values of ACTH, α-MSH, and β-MSH were 0.246 ± 0.048, 0.955 ± 0.262, and 0.421 ± 0.190 nM, respectively (Table 2).

In the presence of tilapia MRAP2b, the potency of ACTH was significantly improved, and its EC_50_ value was reduced to 0.032 ± 0.005 nM. In contrast, the EC_50_ values of α-MSH (0.786 ± 0.162 nM) and β-MSH (0.572 ± 0.122 nM) showed little or no significant change (Table 2).

These findings clearly indicate that tilapia MC4R can be activated efficiently by α-MSH, β-MSH, and ACTH and can act as a common receptor for three peptides. However, in the presence of MRAP2b, MC4R can act as an ACTH-preferring receptor, as previously reported in zebrafish, chickens, and pigs [3,5,21].

**Figure 6:**
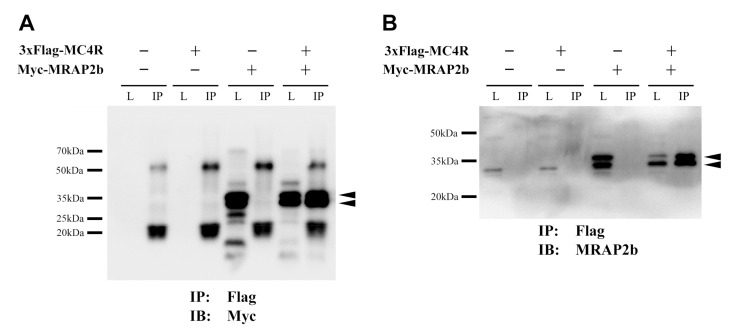
Interaction between tilapia MRAP2b and MC4R. Co-IP assays show the interactions between tilapia MRAP2b and MC4R expressed in CHO cells detected by different antibodies (**A**: anti-Myc-tag; **B**: anti-MRAP2b). Arrow heads indicate the positive tilapia MRAP2b bands (≈35 kDa) in immunoprecipitated samples (IP) detected by the anti-Myc-tag (**A**) or anti-MRAP2 antibody (**B**). The ratio of *MC4R* to *MRAP2b* plasmid used is 1:5 (*w*/*w*). L, cell lysates; IP, immunoprecipitated samples (Anti-Flag antibody used); IB, immunoblotting (anti-Myc-tag antibody/anti-MRAP2 antibody used). *Note*, the two MRAP2b bands (marked by arrowheads) detected are likely caused by *N*-glycosylation at the *N*-terminus (Figure 2).

To confirm the interaction between tilapia MRAP2b and MC4R, Co-IP assay was used in this study. In CHO cells expressing pcDNA3.1-Myc-MRAP2b and pcDNA3.1-3xFlag-MC4R plasmids, magnetic beads immobilized with anti-Flag protein were used to test whether MC4R could interact with MRAP2b. As shown in Figure 6, two positive bands (30–35 kDa) of MRAP2b protein were detected in IP samples co-expressing tilapia MC4R and MRAP2b, supporting the interaction between the two proteins.

### 2.3. AgRP Can Act as an Inverse Agonist of Tilapia MC4R

It was reported that MC4R displays a basal constitutive activity and AgRP can act as an inverse agonist to inhibit this basal constitutive activity in mammals, birds, and zebrafish [3,5,29]. To investigate whether AgRP can inhibit the basal activity of tilapia MC4R, using a dual-luciferase reporter system [5], we analyzed the inhibitory action of recombinant human AgRP on the basal constitutive activity of tilapia MC4R in the absence or presence of MRAP2b.

**Figure 7:**
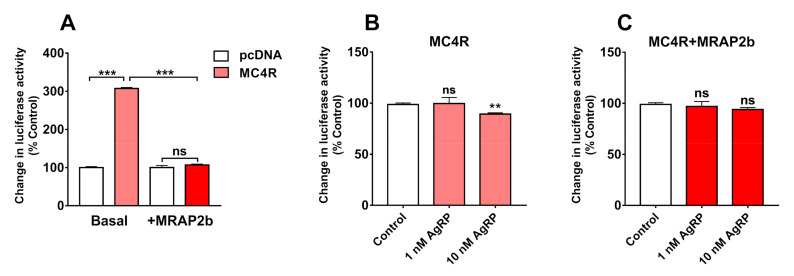
The action of MRAP2b on the basal constitutive activity of tilapia MC4R, as detected by dual-luciferase reporter assay. (**A**) The basal constitutive activity of tilapia MC4R was observed compared to pcDNA3.1 (+) vector control; however, its basal constitutive activity was reduced in the presence of tilapia MRAP2b. (**B**,**C**) The effect of human AgRP (1 nM and 10 nM, 6 h) on the basal luciferase activities of CHO cells expressing tilapia MC4R (**B**), or CHO cells co-transfected with MC4R and MRAP2b plasmids at a ratio of 1:5 (**C**), as monitored by dual-luciferase reporter assay. Data are shown as mean ± SEM of four replicates (*n* = 4). **, *p* < 0.01. ***, *p* < 0.001. ns, no significant difference.

As shown in Figure 7, tilapia MC4R displays a strong basal constitutive activity and induces a 4-fold increase in the luciferase activity of CHO cells expressing MC4R; however, its basal activity could be significantly inhibited by the co-expression of tilapia MRAP2b.

In addition, 10 nM of human AgRP could weakly, but significantly, inhibit the basal activity of MC4R in the absence of MRAP2; however, this inhibitory effect was attenuated by the presence of MRAP2b. This finding indicates that AgRP could act as an inverse agonist for tilapia MC4R (Figure 7).

### 2.4. AgRP Can Antagonize ACTH/α-MSH Actions on MC4R

**Figure 8:**
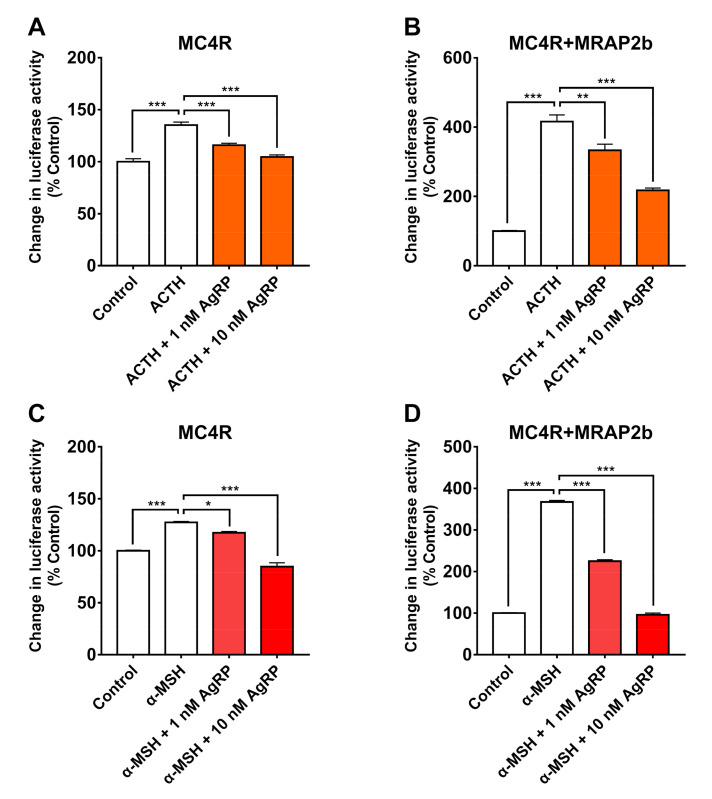
AgRP antagonizes the actions of tilapia ACTH/α-MSH on MC4R in the absence or presence of MRAP2b. (**A**–**D**) Human AgRP (1 nM and 10 nM, 6 h) could dose-dependently block the ACTH (10 nM)-stimulated (**A**,**B**) or α-MSH (10 nM)-stimulated (**C**,**D**) luciferase activity of CHO cells expressing MC4R in the absence (**A**,**C**) or presence (**B**,**D**) of MRAP2b, as monitored by a pGL3-CRE-luciferase reporter system. Each data point represents the mean ± SEM of 4 replicates (*n* = 4). * *p* < 0.05; ** *p* < 0.01; ***, *p* < 0.001.

To determine whether AgRP can antagonize the action of α-MSH or ACTH on tilapia MC4R in the presence or absence of MRAP2b, tilapia MC4R was expressed alone or co-expressed with MRAP2b in CHO cells; then, it was investigated by co-treatment with 10 nM tilapia α-MSH/ACTH and human AgRP (1 nM and 10 nM). As shown in Figure 8, AgRP could block the effect of α-MSH or ACTH on tilapia MC4R dose-dependently, either in the absence or presence of MRAP2b.

### 2.5. Tissue Expression of MC4R, MRAP2b, POMCs, and AgRPs in Tilapia

**Figure 9:**
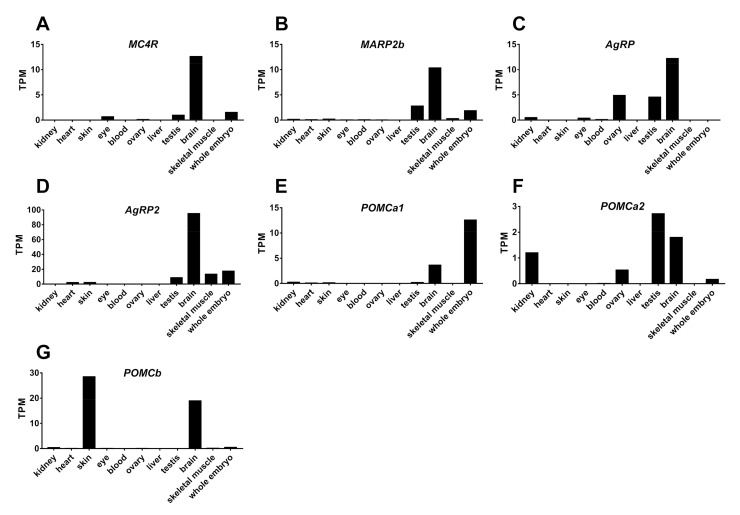
RNA-seq analysis of *MC4R*, *MRAP2b*, *AgRP*, *AgRP2*, *POMCa1*, *POMCa2,* and *POMCb* expression in tilapia tissues. (**A**–**G**) The expression levels of *MC4R* (**A**), *MRAP2b* (**B**), *AgRP* (**C**), *AgRP2* (**D**), *POMCa1* (**E**), *POMCa2* (**F**), and *POMCb* (**G**) mRNA were estimated using the RNA-Seq data (Bioproject PRJNA255848, INRA, France) from tilapia tissues including the kidneys, heart, skin, eyes, blood, ovaries, liver, testes, brain, skeletal muscle, and whole embryos. All data were calculated as TPM (transcript per million).

To examine the tissue expression of the MC4R signaling system in tilapia, we analyzed the transcriptomic data of tilapia tissues (kidneys, heart, skin, eye, blood, ovaries, liver, testes, brain, skeletal muscle, and whole embryos) submitted to the SRA Library of NCBI Database. As shown in Figure 9, all the genes of the MC4R signaling system (*MC4R*, *MRAP2b*, *AgRP*, *AgRP2*, *POMCa1*, *POMCa2*, and *POMCb*) were found to be abundantly expressed in brain tissue. Interestingly, we also found the mRNA expression of some of these genes in the testes (*MC4R*, *MRAP2b*, *AgRP*, *AgRP2*, and *POMCa2*), whole embryos (*MC4R*, *MRAP2b*, *AgRP2*, and *POMCa1*), ovaries (*AgRP*, *POMCa2*, and *MC4R*), eyes *(MC4R* and *AgRP*), kidneys (*AgRP* and *POMCa2*), skin (*AgRP2* and *POMCb*), skeletal muscle (*AgRP2*), and heart (*AgRP2*) (Figure 9).

### 2.6. Fasting Induces Hypothalamic AgRP Expression in Growing Tilapia

**Figure 10:**
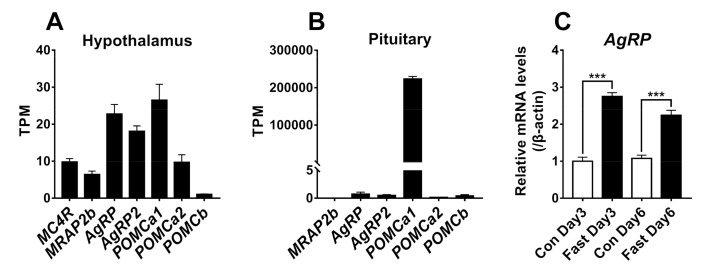
*MC4R*, *MRAP2b*, *AgRP*, *AgRP2*, *POMCa1*, *POMCa2,* and *POMCb* expression in tilapia hypothalamus and pituitary tissues. (**A**) The expression levels of *MC4R*, *MRAP2b*, *AgRP*, *AgRP2*, *POMCa1*, *POMCa2,* and *POMCb* mRNA were estimated using the RNA-Seq data from the hypothalamus of growing tilapia (30-day-old, *n* = 5). RNA-seq data were calculated as TPM (transcript per million). (**B**) The expression levels of *MRAP2b*, *AgRP*, *AgRP2*, *POMCa1*, *POMCa2,* and *POMCb* in the pituitary were estimated using the RNA-Seq data from the pituitary of 30-day-old tilapia (*n* = 2). (**C**) Quantitative real-time RT-PCR assay of *AgRP* expression in the hypothalamus of 30-day-old tilapia under 3-day (Fast day3) or 6-day fasting (Fast day6) compared with that of control (Con day3, Con day6) (i.e., tilapia with free access to food). White pillars and black pillars indicated the control group and fasting group, respectively. Each data point represents the mean ± SEM of four tilapia (*n* = 4). ***, *p* < 0.001 vs. control (Con).

Since all the genes of the MC4R signaling system are expressed in the brain, we further analyzed the RNA-seq data of the hypothalamus from growing tilapia (body weight: ≈38 g). As shown in Figure 10, all the genes are expressed in the hypothalamus. Moreover, we found that 3-day or 6-day fasting could increase the mRNA levels of *AgRP* by more than 2-fold (Figure 10), as detected by quantitative real-time RT-PCR.

## 3. Discussion

The MC4R signaling system has long been proposed to play important roles in the energy balance of teleosts, particularly in the regulation of appetite and growth [19,30,31]. However, the expression and functionality of this MC4R signaling system have not been fully characterized in teleosts. Here, we cloned the genes of the MC4R signaling system and characterized their interaction and expression. Moreover, we found that fasting could significantly induce *AgRP* expression in the hypothalamus. Our data supports the notion that the MC4R signaling system functions in teleosts in a way analogous to that in mammals and birds [2,3,5].

### 3.1. Identification of MC4R Signaling System in Nile Tilapia

Although *MC4R, MRAP2, AgRPs,* and *POMCs* have been predicted to exist in the tilapia genome, the cDNAs of most genes have not been cloned [23,32,33]. In this study, we cloned the coding regions of the six genes from the tilapia brain.

Interestingly, three *POMC* paralogs (named *POMCa1*, *POMCa2*, and *POMCb*) have been identified, similar to previous reports in other ray-finned fish, such as barifin flounder, cichlid fish, and sea bream [22,23,34,35]. As in other teleosts, three tilapia POMC precursors are likely capable of producing three bioactive peptides, namely α-MSH, β-MSH, and ACTH; however, they cannot produce ɤ-MSH present in lungfish, paddlefish, sturgeons, and tetrapods [36,37,38,39]. Moreover, similar to human POMC, tilapia POMCa1 and POMCa2 are likely capable of producing β-endorphins [7,33]. Interestingly, unlike POMCa1 and POMCa2, tilapia POMCb can produce an additional ε-MSH (SYRMEHFRWGKAP), which is also present in the cichlid POMC precursor [23], but it cannot produce a bioactive β-endorphin (which lacks the ‘YGFFM’ motif crucial for the bioactivity of opioid peptides) (Table 1) [7,40]. It was reported that the tissue-specific processing of the POMC precursor by prohormone convertases (PCs) could produce distinct bioactive peptides in target tissues. For instance, in mammals, under the endoproteolytic processing of PC1/3, the POMC precursor can produce mainly ACTH and a minor portion of β-endorphin in pituitary corticotrophs, whereas under the processing of PC2 (and PC1/3), the POMC precursor can produce α-MSH, β-MSH, and β-endorphin as the end products in the hypothalamus and intermediate pituitary [7]. Therefore, the presence of multiple tilapia POMCs also raises an open question of whether all the bioactive peptides listed in Table 1 can be produced after post-translational processing in vivo.

In addition to *POMCs*, we also identified and cloned two *AgRPs* (*AgRP* and *AgRP2*) from the tilapia brain, similar to the findings in other teleosts (e.g., zebrafish, sea bass, and medaka). Synteny analysis revealed that tilapia *AgRP* is orthologous to *AgRP* of humans, chickens, and zebrafish, and it shares a high amino acid sequence identity to human AgRP (Supplementary Materials Figure S1) [24], while *AgRP2* seems to exist in teleosts, but it is likely lost in tetrapods, as previously proposed [13]. Although the amino acid sequence identity shared between the two AgRPs is low (23.4% identity), the cysteine knot structure essential for their confirmation and biological functions are fully conserved in the two proteins, indicating that the two *AgRPs* are likely originated from an ancestral gene [13].

In this study, we also cloned *MC4R* and *MRAP2b* from tilapia brain. Synteny analysis showed that both genes are orthologous to the *MC4R* and *MRAP2* of humans, chickens, and zebrafish (*MRAP2b*) [4,5]. Sequence comparison revealed that MC4R is extremely conserved across vertebrates, particularly in the transmembrane regions. Although MRAP2b shows a high degree of variation in its cytoplasmic tail, the transmembrane domain and ‘LKAHRYS’ motif for anti-parallel homodimer formation are highly conserved across vertebrates [9]. The cloning of these genes confirms the presence and expression of the MC4R signaling system in Nile tilapia, as previously reported in chickens and mammals [5].

### 3.2. The Functionality of the Tilapia MC4R Signaling System

The presence of all MC4R signaling components in tilapia led us to further examine their interaction *in vitro*. We elucidated that in the absence of MRAP2b, tilapia MC4R could be potently activated by tilapia ACTH_1-40_, α-MSH, and β-MSH and stimulate the cAMP/PKA signaling pathway, as monitored by pGL3-CRE-luciferease reporter assay. This finding indicates that as in chickens, ACTH, α-MSH, and β-MSH can function as the ligands for tilapia MC4R [5]. Our finding partly agrees with recent findings in spotted gar, tilapia, topmouth culter, and zebrafish, showing that MC4R could be activated by mammalian α-MSH (or NDP-α-MSH) and ACTH_1-24_/ACTH_1-39_ with similar potencies [3,15,41,42]. Interestingly, in the presence of MRAP2b, the sensitivity and selectivity of MC4R for ACTH_1-40_ increase by ≈8-fold (EC_50_: 0.246 nM vs. 0.032 nM), while its sensitivity to α-MSH and β-MSH shows little or no change (Table 2). This finding clearly indicates that MC4R can interact with MRAP2b, and thus, it acts as an ‘ACTH-preferring receptor’ in tilapia. Our finding contrasts that from a recent study in tilapia, in which MRAP2b cannot significantly alter the EC_50_ value of ACTH_1-39_ in activating tilapia MC4R when expressed in HEK293 cells [41]. The reason for this discrepancy is unknown. However, our finding is consistent with the findings in chickens [5], zebrafish [3], and humans [43], in which MC4R becomes an ACTH-preferring receptor in the presence of MRAP2. Taken together with our co-IP assay results showing the interaction of tilapia MRAP2b with MC4R in vitro (Figure 6), our findings strongly suggest that tilapia MC4R can interact with MRAP2b and act as an ‘ACTH-preferring receptor’.

It was reported that MC4R has a high basal constitutive activity in chickens and mammals [1]. In this study, we proved that tilapia MC4R also has a strong basal constitutive activity, as monitored by dual-luciferase reporter assay. Moreover, we found that MRAP2b can inhibit the basal constitutive activity of tilapia MC4R, as recently reported in orange-spotted grouper, topmouth culter, and tilapia [15,41,42].

In mammals and chickens, AgRP has been demonstrated to function either as an antagonist to block the action of α-MSH and/or ACTH on MC4R, or as an inverse agonist to inhibit the basal activity of MC4R [1,5]. Here, we proved that as in chickens [5], AgRP can dose-dependently block the action of α-MSH and ACTH_1-40_ on tilapia MC4R activation in the presence or absence of MRAP2b. Moreover, AgRP can inhibit the basal activity of tilapia MC4R, and this inhibitory effect diminishes in the presence of MRAP2b. All these findings confirm that as in mammals and birds, AgRP can function as both the antagonist and inverse agonist of MC4R in tilapia [1,5,41]. Since we have no recombinant tilapia AgRP2 in our hands, it remains unclear whether tilapia AgRP2 has a function similar to that of AgRP. However, there is a piece of evidence showing that AgRP2 functions as a potent antagonist of melanocortin receptor 1 (MC1R) in zebrafish [25]. Considering the high structural similarity shared between tilapia and zebrafish AgRP2 (45% sequence identity), it implies that similar to that in zebrafish, the action of AgRP2 is likely mediated by MC1R in tilapia [25].

### 3.3. Tissue Expression of MC4R Signaling System in Tilapia

Using the transcriptomic data of 10 tilapia tissues and whole embryos deposited in the public database, we analyzed the tissue expression of the MC4R signaling system and found that the expression of *MC4R*, *MRAP2b*, *AgRP*, *AgRP2*, *POMCa1*, *POMCa2*, and *POMCb* is restricted to the brain and several extra-brain tissues (e.g., gonads) (Figure 9).

**Figure 11:**
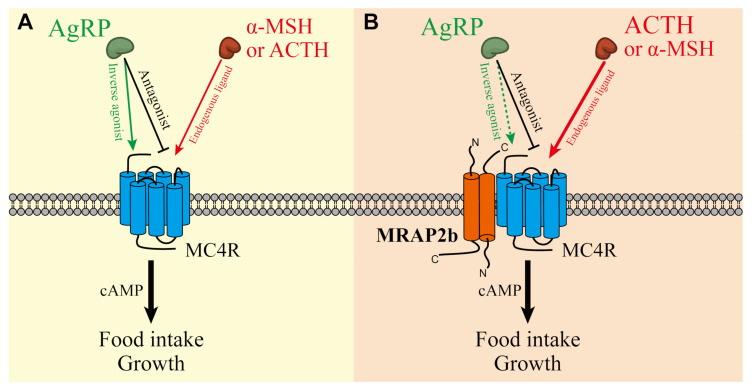
MC4R signaling system in tilapia. (**A**) In the absence of MRAP2b, α-MSH and ACTH can activate MC4R, while AgRP can act as an antagonist to block the action of α-MSH and ACTH. AgRP alone can also act as an inverse agonist to block the constitutive activity of MC4R. (**B**) In the presence of MRAP2b, MC4R interacts with MRAP2b and thus decreases the basal constitutive activity of MC4R and enhance its sensitivity and selectivity for ACTH. AgRP seems to mainly act as an antagonist to block the action of ACTH/α-MSH on MC4R activation. The interaction between these molecules in the hypothalamus determines MC4R signaling intensity, which may affect the feeding and growth in tilapia and other vertebrate species (e.g., zebrafish, chickens, pigs).

In the brain, all genes were detected to be highly or moderately expressed. This finding is consistent with previous reports in teleosts, such as zebrafish [3] and medaka [6], in which all the genes are expressed in the brain as detected by qRT-PCR assays. Furthermore, we found that all the genes are expressed in the hypothalamus of growing tilapia, suggesting that the central MC4R signaling system plays roles in the control of feeding and energy balance in tilapia. In agreement with this speculation, we proved that 3-day or 6-day fasting causes a > 2-fold increase of *AgRP* expression in tilapia hypothalamus, suggesting AgRP may play a role in the regulation of appetite and energy balance. Our observation is consistent with the findings in zebrafish [24], sea bass [44], and goldfish [31] in which fasting increases *AgRP* expression in their hypothalamus. In addition, the injection of MC4R antagonists (HS024) can stimulate food intake in goldfish and rainbow trout [30,45], and ip/icv injection of MC4R agonists (ACTH, MTII) can reduce the feeding in zebrafish [3], goldfish [30], and rainbow trouts [45]. Taken together, these evidence led us to hypothesize that as in zebrafish, AgRP and ACTH/α-MSH competitively act on the MC4R or MC4R-MRAP2 complex, to regulate feeding and growth in Nile tilapia (Figure 11) [3]. Although two *AgRPs* are expressed in the hypothalamus of teleosts such as zebrafish and tilapia, only *AgRP* has been reported to be expressed in the zebrafish/goldfish hypothalamic lateral tuberal nucleus, which is a structure homologous to mammalian arcuate nucleus [31]. Since AgRP2 has been proven to be a potent antagonist of MC1R (not MC4R) in zebrafish [25], the central action of AgRP2 seems unlikely to be mediated by MC4R.

Outside the brain, *POMCa1* is predominantly expressed in tilapia pituitary (Figure 10), while other components of the MC4R system show little or no expression. This finding differs from those in barfin flounder and gilthead sea bream, in which both *POMCa1* and *POMCa2* are abundantly expressed in pituitary melanotrophs and corticotrophs [34,46,47]. The predominant expression of *POMCa1* in tilapia pituitary suggests that ACTH and α-MSH derived from the POMCa1 precursor can be released from corticotrophs and melanotrophs respectively and act on peripheral tissues via an endocrine route, such as the regulation of skin pigmentation and inter-renal cortisol production [48].

Of particular interest to note is that *POMCb* is highly expressed in the skin. Similarly, *POMCb* is also expressed in the skin of barfin flounder [35,48]. In addition, α-MSH/ACTH immunoreactivity was detected in the germinal layer of African lungfish skin epidermis [49]. These findings support the hypothesis that α-MSH/ACTH can be synthesized by the skin; thus, skin pigmentation is likely controlled by α-MSH/ACTH produced from the pituitary (e.g., *POMCa1*) and skin (*POMCb*) in teleosts [48].

In this study, we found that *MC4R* is expressed in tilapia gonads, including the testes and ovaries. Moreover, tilapia *AgRP*, *POMCa1*, and *MRAP2b* were found to be expressed in the testes and *AgRP* and *POMCa2* were found to be expressed in the ovaries (Figure 9). This concurs with previous findings in other fish, such as the expression of *MC4R*, *MRAP2b*, *AgRP*, and *POMCb* in medaka gonads [6], and *MC4R* in the testes and/or ovaries of barfin flounder [50], sea bass [51], and goldfish [30]. Taken together, these findings support that MC4R and their ligands (either derived locally or from the pituitary) play important roles in gonadal development and functions in teleosts, such as the stimulation of testosterone production in the testes, as previously reported in mice [52]. In addition, we found a weak expression of *MC4R* and *AgRP* in the eyes, and *POMCa1*, *POMCa2*, and *POMCb* in the kidneys. The question of whether MC4R signaling plays a role in these tissues remains to be clarified.

Apart from the expression of *MC4R* in adult tilapia tissues, *MC4R, MRAP2b,* and *POMCa1* are expressed in whole embryos (Figure 9). Similarly, *MC4R* and *MRAP2* are expressed at the late neurula stage and early stage of brain development, and *POMCa1* is expressed two days later than *MC4R* in medaka embryos. Moreover, gene knockout of *MC4R* delays hatching [6]. These findings hint that MC4R signaling plays important roles in the embryonic development of teleosts (e.g., brain development), even in the absence of its ligands. Future study on this topic would provide more clues on this issue.

In summary, we cloned six genes of the MC4R signaling system in a representative teleost species, Nile tilapia. Functional study proved that MC4R can interact with MRAP2b and mediate the actions of α-MSH, ACTH, and AgRP in vitro. Moreover, we demonstrated that MRAP2b cannot only inhibit the basal constitutive activity of MC4R, but also increase its sensitivity and selectivity for ACTH, thus becoming an ACTH-preferring receptor. These findings, together with evidence showing the co-expression of *MC4R*, *POMCs*, *AgRP,* and *MRAP2b* in the hypothalamus, strongly suggest that the MC4R signaling system plays a conserved role in the regulation of food intake and growth in Nile tilapia (Figure 11). Considering that Nile tilapia is a freshwater fish of economic importance worldwide, our data provides a theoretical basis to improve the economic traits of tilapia, such as promoting the appetite and growth via modifying MC4R signaling (e.g., gene-editing MC4R system) in aquaculture.

## 4. Materials and Methods

### 4.1. Chemicals, Primers, Peptides, and Antibodies

All chemicals were purchased from Sigma-Aldrich, and restriction enzymes were obtained from TaKaRa. Plasmid vector pTA2 used for the T/A cloning of PCR products was purchased from TOYOBO Company. The eukaryotic expression vector is pcDNA3.1 (+) purchased from Invitrogen (Carlsbad, CA, USA). Tilapia (ti-) ACTH_1-40_, α-MSH (acetyl-α-MSH with an amidated C-terminus), and β-MSH were synthesized by GL Biochem Ltd. (Shanghai, China). The purity of synthesized peptides is more than 95% (analyzed by HPLC), and their structures were verified by mass spectrometry. Recombinant human AgRP protein (Cat no. 704-AG) was purchased from R&D Systems. RNAzol was purchased from the Molecular Research Center (Cincinnati, Ohio, USA). Anti-Flag Affinity Gel beads were purchased from Bimake (Bimake, Houston, TX, USA). Rabbit anti-MRAP2 antibody (612249) and mouse anti-Myc-tag antibody (R950-25) were obtained from Chengdu Zhengneng Company (Zhengneng, Chengdu, China) and Thermo Scientific (Asheville, NC, USA), respectively. All the primers used in this study were synthesized by Chengdu Qingke and listed in Table S1.

### 4.2. Animals, Tissues, and Ethical Statement

Tilapia (GIFT, Genetic Improvement of Farmed Tilapia, *Oreochromis niloticus*, ♂) were purchased from the Chongqing Freshwater Aquaculture Farm (Chongqing, China). Tilapia (weight 35–50 g) were cultured in the experimental culture system of the College of Life Sciences, Sichuan University. Fish were anesthetized with buffered MS-222 (Sigma-Aldrich, St. Louis, MO, USA) and then dissected. The tissues (hypothalamus and pituitary) were collected, quickly frozen in liquid nitrogen, and stored at −80 °C until use. All animal experimental protocols used in this study were approved by the Animal Ethics Committee of College of Life Sciences, Sichuan University, and the assurance number is 20200902001 (2 September 2020).

### 4.3. Total RNA Extraction and Reverse Transcription (RT)

Total RNA was extracted from tilapia tissues using RNAzol and dissolved in DEPC-treated H_2_O. These RNA samples were reversely transcribed by Moloney murine leukemia virus (MMLV) reverse transcriptase (Takara) and were used for the PCR amplification of target genes as described in our recent study [53].

### 4.4. Cloning of Tilapia MC4R, MRAP2, POMCs, and AgRPs

With reference to the cDNAs of human and zebrafish *MC4R, MRAP2, POMCs,* and *AgRP*, we searched the tilapia genome (http://www.ensembl.org/Oreochromis_niloticus) and identified the DNA fragments of the above genes. Then, the primers were designed based on this information. The cDNAs from tilapia brain tissue were used as a template for PCR amplification, and the PCR products were cloned into pTA2 vector (TOYOBO, Japan) and sequenced by the Beijing Genome Institute (BGI). Finally, the coding regions of tilapia *MC4R, MRAP2* (named *MRAP2b* in this study)*, POMCs*, and *AgRPs* were determined.

### 4.5. RNA-Seq Analysis of MC4R, MRAP2b, POMCs, and AgRPs in Tilapia Tissues

The RNA Seq data (BioProject: PRJNA78915) obtained by Brawand et al. were used to analyze the expression of the MC4R signaling system in adult tilapia [54]. RNA-Seq data were downloaded from the NCBI website (http://www.ncbi.nlm.nih.gov/sra). With reference to the Orenil 1.0 transcription group (Ensembl 94 edition), we quantified the gene expression level using the transcript quantitative analysis tool Salmon v0.10.2 and default parameters. The relative abundance of tilapia *MC4R*, *MRAP2b*, *POMCa1*, *POMCa2*, *POMCb*, *AgRP,* and *AgRP2* transcripts are expressed as the transcripts per million (TPM) of each million reads.

The transcriptome of the hypothalamus and pituitary of growing tilapia (≈40 g body weight) available in our lab was also studied using the same approach as above.

### 4.6. Quantitative Real-Time RT-PCR Assay (qRT-PCR)

To determine whether fasting can alter hypothalamic *AgRP* expression, 80 tilapia (*♂*) of uniform bodyweight (37–38 g) at the growing stage were divided into four groups: two control groups and two fasting groups. After fasting for 3 days and 6 days, the hypothalamus of tilapias were collected for total RNA extraction and subjected to quantitative real-time RT-PCR assay of *AgRP* expression, as described in our recent study [5,53].

### 4.7. Functional Analysis of tiMC4R and tiMRAP2b

Based on the cloned cDNAs of tilapia *MC4R* and *MRAP2b*, the coding regions of these genes were amplified by PCR with high-fidelity Taq DNA polymerase and cloned into pcDNA3.1 (+) vector. According to our established method, tilapia MC4R transiently expressed in CHO cells was treated by synthetic tilapia ACTH/α-MSH/β-MSH (10^−10^ to 10^−9^ M, 6 h), and then, the receptor-activated cAMP signaling pathway was monitored by a pGL3-CRE-Luciferase reporter system, which is capable of monitoring the receptor-mediated cAMP level [28,55]. To test whether tilapia MRAP2b could alter the pharmacological property of tilapia MC4R, CHO cells co-transfected with MRAP2 and receptor expression plasmids were treated by tilapia ACTH, α-MSH, and β-MSH, and the relative potencies of the three peptides in activating MC4R were evaluated by the mentioned reporter system [28,55].

### 4.8. Co-Immunoprecipitation (Co-IP) Assay

To investigate the interaction of tilapia MC4R with MRAP2, we first prepared the pcDNA3.1 (+) expression plasmids encoding an N-terminally Myc-tagged MRAP2b (pcDNA3.1-Myc-MRAP2b) and N-terminally Flag-tagged MC4R (pcDNA3.1-3XFlag-MC4R) [5]. The Co-IP assay was carried out as described in our previous study [5]. Then, Western blot was performed with mouse anti-Myc-tag antibody (1:2000) and rabbit anti-MRAP2 antibody (1:1000).

### 4.9. Detection of the Inverse Agonistic and Antagonistic Actions of AgRP

To evaluate whether AgRP could act as an inverse agonist to inhibit the basal constitutive activity of MC4R, or act as an antagonist to block the action of α-MSH/ACTH on MC4R activation, CHO cells transfected with tilapia MC4R expression plasmid, or co-transfected with MC4R and MRAP2 expression plasmid, were treated by recombinant human AgRP (1 nM and 10 nM) alone, or by both human AgRP (1nM and 10 nM) and tilapia α-MSH/ACTH(10 nM). Then, the receptor-mediated cAMP production was monitored by the pGL3-CRE-luciferase reporter system, as described in our recent study [5].

### 4.10. Data Analysis

The relative mRNA levels of hypothalamic *AgRP* were first normalized by *β-actin* and then expressed as a fold difference compared with their respective controls. The reporter assay data were analyzed by one-way ANOVA followed by Dunnett’s test. The dose–response curves were constructed using nonlinear regression models, and the corresponding half-maximal effective concentration (EC_50_) values were evaluated with Graphpad Prism 7 (GraphPad Software, San Diego, CA, USA). All experiments were repeated at least twice to validate our results.

## Figures and Tables

**Table 1:** Amino acid sequences of peptides derived from three tilapia POMC precursors.

Peptide	Gene	Amino Acid Sequence
ACTHs	*POMCa1*	SYSMEHFRWGKPVGRKRRPVKVYTSNGVAEESAEVFPEEM
	*POMCa2*	SYSMEHFRWGKPVGRKRRPIKIYTTNGLEEESAELFPGEM
	*POMCb*	SYSMEHFRWGKPSGRKRRPVKVFASSLEGGSSSEGRFPFQ
α-MSHs	*POMCa1*	SYSMEHFRWGKPV
	*POMCa2*	SYSMEHFRWGKPV
	*POMCb*	SYSMEHFRWGKPS
β-MSHs	*POMCa1*	DGSYKMKHFRWSGPPAS
	*POMCa2*	DEMYKMKHFRWGGLPAS
	*POMCb*	DRTYKMSHFRWGSPPAS
ε-MSH	*POMCb*	SYRMEHFRWGKPA
β-endorphins	*POMCa1*	YGGFMKSWDERSQRPLLTLFKNVINKEGQQQK
	*POMCa2*	YGGFMKSWDERSQRPLITLFKNVINKEAQEEKREQ
	*^a^* *POMCb*	NGGFMKPWEEKPQGQLAKFFRNILVKDVKRIMG

Note: ‘a’ indicates that β-endorphin derived from the POMCb precursor may not be a bioactive peptide (due to loss of a ‘YGGFM motif’).

**Table 2:** EC_50_ values of ACTH, α-MSH, and β-MSH in activating the cAMP/PKA signaling pathway in CHO cells expressing tilapia MC4R in the absence or presence of MRAP2b.

Peptides	EC_50_ (nM)	
	MC4R	MC4R + MRAP2b
ACTH	0.246 ± 0.048	0.032 ± 0.005
α-MSH	0.955 ± 0.262	0.786 ± 0.162
β-MSH	0.421 ± 0.190	0.572 ± 0.122

## Supplementary Materials

The following are available online at https://www.mdpi.com/1422-0067/21/19/7036/s1.
