# Supplementary material for: Melanocortin Receptor 4 (MC4R) Signaling System in Nile Tilapia
Source: Int J Mol Sci. 2020 Sep 24;21(19):7036. doi: 10.3390/ijms21197036 (PMC7582737; doi:10.3390/ijms21197036)
Supplement: Supplementary file 1 [file ijms-21-07036-s001.pdf]

Supplementary Table 1. Primers used in this study[a].

Gene	Sense/antisense	Primer sequence (5′-3′)
	Primers for constructing expression plasmids or cloning	
MC4R	Sense	CGGAATTCGCCAGCATGAATGCCACAGAATACCA
	Antisense	CGGAATTCTCACACATACAGCAGAGCAT
MRAP2b	Sense	CGGAATTCGCCAGCATGCGCACTGAGAAGCCCCC
	Antisense	CGGAATTCTTAGTGGATGTCAAAGTGAG
POMCa1	Sense	ATGTGTCCTGTGTGGCTTTT
	Antisense	TCACTTTTGCTGCTGTCCTT
POMCb	Sense	ATGGTGTGCCAGTGCTGGTT
	Antisense	TCATCCCATTATTCTCTTCACATCT
AgRP	Sense	ATGAGGCTCTTGTTGGAGAA
	Antisense	CAGCAGCTTGTTATCAGGTG
AgRP2	Sense	ATGAGGAAGATCACCGGCAA
	Antisense	CTAGGTCCTCTTTAAGCAGA
	Primers for quantitative Real-time PCR assay	
AgRP	Sense	ATGAGGCTCTTGTTGGAGAA
	Antisense	CAGCAGCTTGTTATCAGGTG
ACTB1	Sense	GATCTGGCATCACACCTTCT
	Antisense	GCCTGGATGGCAACGTACAT

[a]All primers were synthesized by Tsingke (Beijing, China). [b]Restriction sites added in the 5′-end of the primers are underlined.

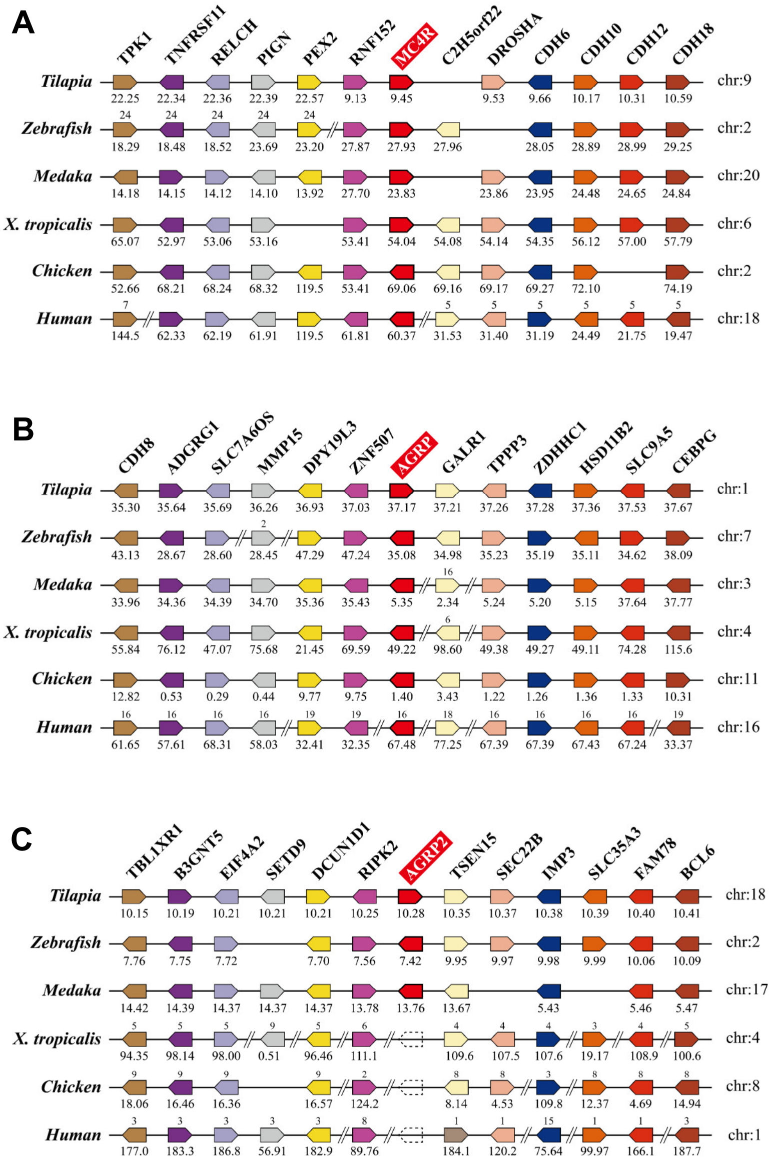

Figure 1. Synteny analysis of *MC4R* (**A**), *AgRP* (**B**), and *AgRP2* (**C**) and their neighboring genes in Nile tilapia, zebrafish, Japanese medaka, western clawed frog (*Xenopus tropicalis*), chickens, and humans. Orthologs are aligned in the same pentagon with the same color. Chromosome (Chr.) numbers are represented above the genes or listed on the right, and the locations (in megabase, Mb) on the chromosomes are shown below the genes based on the information from ENSEMBL databases. Note: *AgRP2* is lost in western clawed frogs, chickens and humans.
